# Efficacy of checkpoint inhibitors in genitourinary cancers with advanced chronic kidney disease

**DOI:** 10.1007/s00262-026-04482-0

**Published:** 2026-07-10

**Authors:** Yin-Shen Chen, Chia-Cheng Yu, Renin Chang, Sheng-Hua Wu, Cheuk-Kwan Sun, Shu-Hung Huang

**Affiliations:** 1 Division of Urology, Department of Surgery, Kaohsiung Veterans General Hospital, No. 386, Dazhong 1 Rd., Kaohsiung City, 813414 Taiwan; 2 School of Medicine, College of Medicine, Kaohsiung Medical University, Kaohsiung, 813414 Taiwan; 3 Department of Medical Education and Research and Department of Emergency Medicine at Kaohsiung Veterans General Hospital, Kaohsiung, 813414 Taiwan; 4 School of Medicine, Chung Shan Medical University, Taichung, 40201 Taiwan; 5 Department of Anesthesiology, Kaohsiung Municipal Ta-Tung Hospital, Kaohsiung, Taiwan; 6 Department of Anesthesiology, School of Medicine, College of Medicine, Kaohsiung Medical University, Kaohsiung, 807378 Taiwan; 7 Department of Emergency Medicine, E-Da Dachang Hospital, I-Shou University, Kaohsiung, 84001 Taiwan; 8 Division of Plastic Surgery, Department of Surgery, Kaohsiung Medical University Hospital, Kaohsiung, 80756 Taiwan; 9 Department of Medical Laboratory Science and Biotechnology, Fooyin University, Kaohsiung, Taiwan; 10 College of Nursing, Mei-Ho University, Pingtung, Taiwan; 11 School of Medicine for International Students, College of Medicine, I-Shou University, Kaohsiung, Taiwan

**Keywords:** Chronic kidney disease, Genitourinary cancers, Immunological checkpoint inhibitors

## Abstract

**Background:**

Immunological checkpoint inhibitors (ICIs) have shown promise in treating various malignancies but are understudied in genitourinary cancers among patients with advanced chronic kidney disease (CKD), who are typically excluded from clinical trials. We evaluated the efficacy and safety of ICIs in this high-risk patient group.

**Methods:**

This retrospective cohort study included patients with CKD diagnosed with renal cell carcinoma and urothelial carcinoma, utilizing data from 63 healthcare organizations in the TriNetX US Collaborative Network database between January 2015 and December 2023. Patients with advanced CKD (aCKD) and early CKD (eCKD) receiving ICIs were compared after 1:1 propensity score matching. Outcomes were assessed using Kaplan–Meier and Cox proportional hazards models. The primary outcome was all-cause mortality, and secondary outcomes included immune-related adverse events (irAEs).

**Results:**

The study involved 2213 patients with aCKD and 9784 with eCKD who received ICIs. After matching, 2196 patients remained in each cohort. Patients with aCKD had higher 2-year all-cause mortality than those with eCKD (44.7% vs. 35.5%; HR = 1.372, 95% CI: 1.248–1.507). They also had a modestly higher risk of overall coded irAEs (HR = 1.141, 95% CI: 1.058–1.231), mainly driven by AKI (HR = 1.662, 95% CI: 1.500–1.840). Increased risks of mortality and AKI were evident from 3 months and persisted through 60 months.

**Conclusions:**

In this large retrospective database study, aCKD was associated with worse survival and greater renal vulnerability among ICI-treated patients with genitourinary cancers. CKD alone should not automatically preclude ICI use in carefully selected patients, but close renal monitoring and multidisciplinary management are warranted, particularly for patients with aCKD.

Trial registration number: Not applicable.

**Supplementary Information:**

The online version contains supplementary material available at https://doi.org/10.1007/s00262-026-04482-0.

## Introduction

Immunological checkpoint inhibitors (ICIs), which are antibodies that boost immune responses, have significantly improved survival for patients with advanced malignancies [1]. As of December 2020, the United States Food and Drug Administration (FDA) had approved six anti-PD-1/PD-L1 antibodies for 19 cancers [2], including renal cell carcinoma (RCC) and urothelial carcinoma (UC). Chronic kidney disease (CKD), defined by impaired kidney function and reduced glomerular filtration rate (GFR), is common in patients with RCC and UC and may arise after nephron loss from surgery or nephrotoxic systemic therapies [3, 4]. Patients with advanced CKD (aCKD), specifically stages 4–5 according to the Kidney Disease: Improving Global Outcomes (KDIGO) guidelines [5], often require renal replacement therapy. However, data on the safety and effectiveness of ICIs in cancer patients with aCKD are scarce because these patients are frequently excluded from major clinical trials [6–9], with limited reports on ICI use in those with poor renal function [10–14].

ICIs reactivate exhausted antitumor T cells in the tumor microenvironment, but uremia has been shown to impair CD4⁺ T-cell activation and proliferation [15, 16], raising concerns that aCKD may attenuate antitumor immune responses. At the same time, ICIs are associated with multisystem immune-related adverse events (irAEs), which can affect up to two-thirds of treated patients [17]. A recent review reported acute kidney injury (AKI) in 5.7% of cancer patients receiving ICIs, with a median onset of 108 days [18]. Patients with RCC and UC exhibit higher AKI risks [19], likely due to greater immune infiltration enhancing irAEs [20]. Nevertheless, observational data suggest that the overall incidence of irAEs in patients undergoing dialysis is broadly comparable to that in the general population [12], leaving the net benefit and risk profile of ICIs in aCKD uncertain.

Given these inconsistencies, the routine exclusion of patients with aCKD from clinical trials [6–9, 21], and lack of real-world evidence, we conducted a retrospective cohort study using the TriNetX US database to compare outcomes of immune checkpoint inhibitor therapy across CKD stages. Specifically, we examined whether patients with aCKD differed from those with early CKD (eCKD) in terms of all-cause mortality and immune-related adverse events.

## Methods

### Data source and study design

The US Collaborative Network section of the TriNetX database, which includes records from 63 US healthcare organizations, provides the framework for this retrospective cohort study, spanning January 2015 to December 2023, with analyses completed in May 2026. The primary aim was to evaluate all-cause mortality and 2-year survival rates associated with ICI therapy in genitourinary cancers, with a focus on the varying CKD stages. The secondary objectives were to assess the impact of cancer type, patient age, and dialysis status on mortality and irAE incidence across different CKD stages. The Institutional Review Board (IRB) of Kaohsiung Veterans General Hospital approved the study, which waived the requirement for informed consent owing to its retrospective observational nature using de-identified data (IRB No. KSVGH24-CT3-09).

### Study population

The index date was defined as the date of the first ICI prescription, with a 14-day washout period to reduce noise from unrelated treatment events. Patients were retrospectively identified and stratified into two groups based on their renal function status within 6 months before the index date. The KDIGO guidelines [5] classify CKD into five stages based on the GFR. aCKD included patients with stage 4 or 5 CKD, end-stage renal disease (ESRD), dialysis-related diagnosis or procedure codes, or any recorded eGFR < 30 mL/min/1.73 m^2^ within 6 months before the index date. eCKD included patients with stage 1 to 3 CKD or eGFR ≥ 30 mL/min/1.73 m^2^ during the same period, with no evidence of eGFR < 30 mL/min/1.73 m^2^, CKD stage 4–5, ESRD, or dialysis. Because patients could have multiple eGFR measurements during the baseline window, TriNetX-generated categorical eGFR indicators reflected any recorded value within each range and were not mutually exclusive. For patients without eGFR measurements, CKD stage was assigned according to ICD-10-CM codes for CKD stages, ESRD, or dialysis. Kidney transplant recipients were excluded owing to potential impact on immune function and ICI efficacy.

### Exclusion criteria

Patients who underwent kidney transplantation, received ICI therapy before cancer diagnosis, or died or experienced irAEs within 14 days of ICI treatment initiation were excluded.

### Case identification and inclusion criteria

We used the International Classification of Diseases, 10th Revision, Clinical Modification (ICD-10-CM) codes to identify RCC (code C64) and UC (renal pelvis [C65], ureter [C66], and bladder cancers [C67]) [22]. ICIs were identified using RxNorm and HCPCS codes for drugs, such as pembrolizumab and nivolumab. IrAEs were identified using validated ICD-10-CM codes for conditions, such as skin toxicity, thyroid dysfunction, colitis, pneumonitis, hepatitis, and AKI (Supplementary Table S1).

### Statistical analysis

We employed the built-in analysis module in TriNetX to compare baseline characteristics between the aCKD and eCKD groups and to perform 1:1 greedy nearest-neighbor propensity score matching (PSM) based on covariates with an absolute standardized mean differences (SMDs) > 0.1 before matching. After matching, an absolute SMD < 0.1 was considered adequate covariate balance. Because renal function was used to define the exposure groups, eGFR categories were not included as PSM covariates. Missing values were handled according to the default TriNetX implementation, and analyses were conducted based on the available data without requiring additional imputation.

Kaplan–Meier methods were used to estimate the cumulative probability of all-cause mortality and irAEs during the 2-year follow-up period, and differences between cohorts were assessed using log-rank tests. Kaplan–Meier curves were re-plotted using SAS version 9.4. Univariate hazard ratios (HRs) with 95% confidence intervals (CIs) at different follow-up durations (3, 6, 12, 24, 36, and 60 months) were generated using the built-in outcome analysis module in TriNetX.

Given that multivariable Cox proportional hazards regression could only be performed in the original pre-matched cohorts in TriNetX, adjusted HRs (aHRs) with 95% CIs were estimated using Cox proportional hazards models in the pre-matched cohorts, adjusting for the same covariates used for PSM. Age-stratified subgroup analyses (< 65 vs ≥ 65 years at index) were conducted using the same analytical approach.

Exploratory Kaplan–Meier subgroup analyses stratified by cancer type (RCC vs UC) and dialysis status (aCKD with dialysis, aCKD without dialysis, and eCKD) were performed in the original sub-cohorts without PSM during the 2-year follow-up period.

### Patient and public involvement statement

Patient and public involvement was not applicable, as this retrospective cohort study utilized de-identified patient data sourced from the TriNetX database. The data were accessed and analyzed in aggregate form without any direct patient contact or individual patient data review.

## Results

### Baseline characteristics

**Fig. 1:**
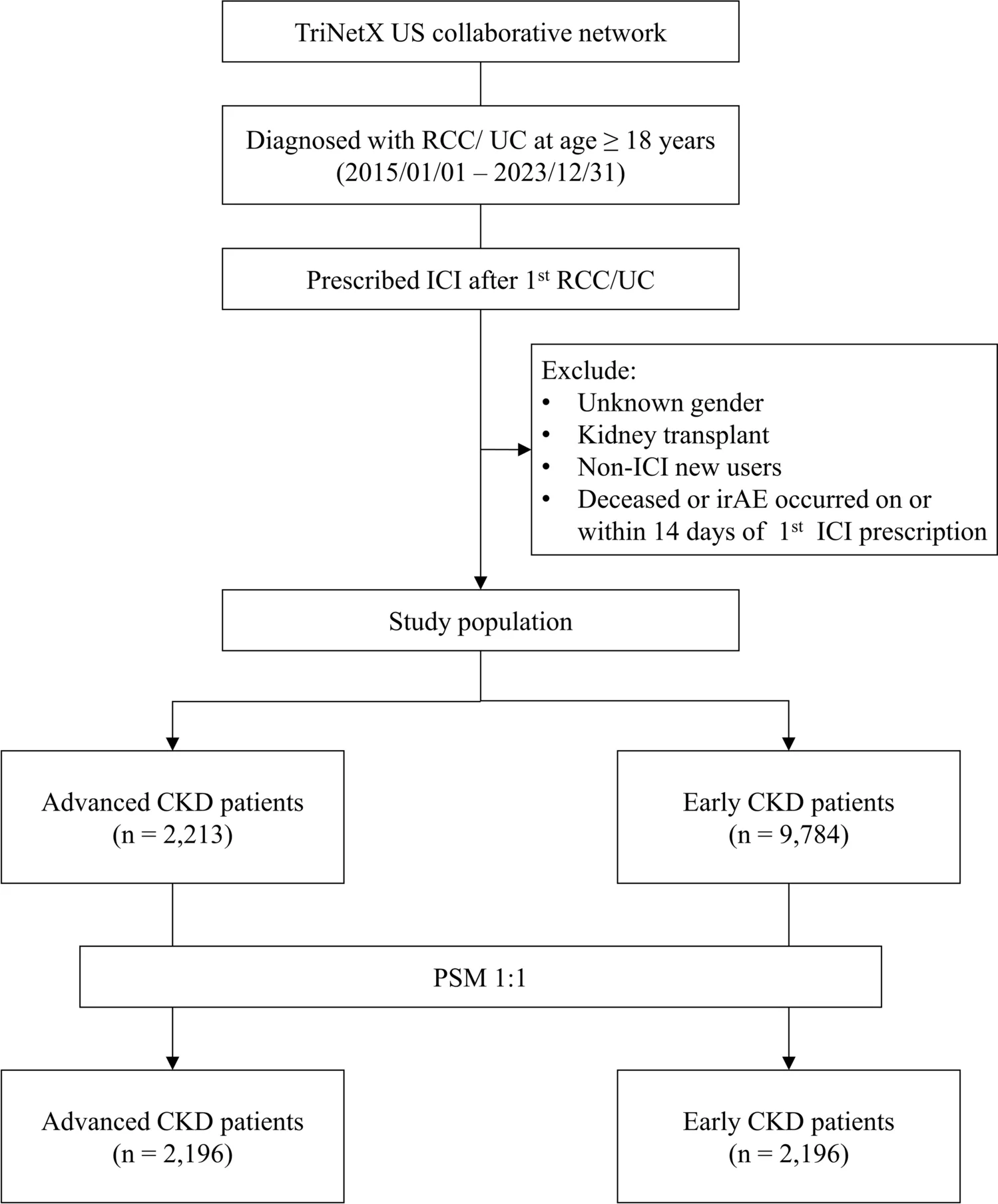
Flow chart of selection

From January 2015 to December 2023, we identified 2213 patients with aCKD (eGFR < 30 mL/min/1.73 m^2^ or CKD stage 4–5) and 9784 with eCKD (GFR ≥ 30 mL/min/1.73 m^2^ or CKD stage 1–3) at ICI initiation (Fig. 1). In the aCKD cohort, ICD-10-CM codes for RCC (C64) were present in 1088 patients (49.2%), while codes for renal pelvic, ureteral, and bladder cancers (C65–C67) were present in 160 (7.2%), 189 (8.5%), and 1096 (49.5%) patients, respectively. In the eCKD cohort, RCC codes were recorded in 5207 patients (53.2%), while codes for renal pelvic, ureteral, and bladder cancers were present in 460 (4.7%), 486 (5.0%), and 3970 (40.6%) patients, respectively. All baseline characteristics are summarized in Table 1 and Supplementary Table S2.

**Table 1:** Baseline characteristics of the study population before and after PSM

	Before PSM			After PSM		
	Patients with aCKD (n = 2213)	Patients with eCKD (n = 9784)	SMD	Patients with aCKD (n = 2196)	Patients with eCKD (n = 2196)	SMD
*Age at index (years)*	69.4 ± 9.9	67.1 ± 10.6	**0.228**	69.4 ± 9.9	69.6 ± 9.6	0.030
*Sex, n (%)*						
Male	1519 (68.6)	7128 (72.9)	0.093	1513 (68.9)	1479 (67.4)	0.033
Female	694 (31.4)	2656 (27.1)	0.093	683 (31.1)	717 (32.7)	0.033
*Race, n (%)*						
White	1737 (78.5)	7894 (80.7)	0.054	1730 (78.8)	1720 (78.3)	0.011
Black or African American	250 (11.3)	774 (7.9)	**0.115**	242 (11.0)	227 (10.3)	0.022
Asian	78 (3.5)	387 (4.0)	0.023	77 (3.5)	92 (4.2)	0.036
American Indian or Alaska Native	11 (0.5)	58 (0.6)	0.013	11 (0.5)	10 (0.5)	0.007
Other races	51 (2.3)	290 (3.0)	0.041	51 (2.3)	62 (2.8)	0.032
Unknown race	72 (3.3)	330 (3.4)	0.007	71 (3.2)	79 (3.6)	0.020
*Comorbidities, n (%)*						
Hypertensive diseases	1514 (68.4)	5106 (52.2)	**0.336**	1497 (68.2)	1471 (67.0)	0.025
Disorders of lipoprotein metabolism and other lipidemias	1021 (46.1)	3483 (35.6)	**0.216**	1013 (46.1)	1000 (45.5)	0.012
Diabetes mellitus	718 (32.4)	2039 (20.8)	**0.265**	706 (32.1)	665 (30.3)	0.040
Ischemic heart diseases	619 (28.0)	1929 (19.7)	**0.195**	611 (27.8)	572 (26.0)	0.040
Chronic lower respiratory diseases	456 (20.6)	1773 (18.1)	0.063	451 (20.5)	480 (21.9)	0.032
Disorders of thyroid gland	410 (18.5)	1481 (15.1)	0.091	401 (18.3)	417 (19.0)	0.019
Diseases of arteries, arterioles and capillaries	420 (19.0)	1322 (13.5)	**0.149**	410 (18.7)	425 (19.4)	0.017
Sleep disorders	362 (16.4)	1285 (13.1)	0.091	356 (16.2)	351 (16.0)	0.006
Depressive episode	247 (11.2)	864 (8.8)	0.078	244 (11.1)	256 (11.7)	0.017
Cerebrovascular diseases	184 (8.3)	627 (6.4)	0.073	181 (8.2)	186 (8.5)	0.008
Malignant neoplasm of kidney, except renal pelvis (C64)	1088 (49.2)	5207 (53.2)	0.081	1078 (49.1)	1054 (48.0)	0.022
Malignant neoplasm of renal pelvis (C65)	160 (7.2)	460 (4.7)	**0.107**	152 (6.9)	165 (7.5)	0.023
Malignant neoplasm of ureter (C66)	189 (8.5)	486 (5.0)	**0.143**	183 (8.3)	190 (8.7)	0.011
Malignant neoplasm of bladder (C67)	1096 (49.5)	3970 (40.6)	**0.181**	1086 (49.5)	1090 (49.6)	0.004
Overweight and obesity	396 (17.9)	1320 (13.5)	**0.121**	391 (17.8)	364 (16.6)	0.033
Acquired absence of kidney	645 (29.1)	2190 (22.4)	**0.155**	632 (28.8)	619 (28.2)	0.013
*Medicines, n (%)*						
Antibacterials for systemic use	1584 (71.6)	5578 (57.0)	**0.308**	1568 (71.4)	1594 (72.6)	0.026
Proton pump inhibitors	763 (34.5)	2430 (24.8)	**0.212**	752 (34.2)	741 (33.7)	0.011
Systemic corticosteroid use	1321 (59.7)	5456 (55.8)	0.080	1307 (59.5)	1409 (64.2)	0.096
*Laboratories, n (%)*						
BMI (kg/m^2^)	28.4 ± 6.9	28.2 ± 6.5	0.025	28.3 ± 6.8	27.9 ± 6.5	0.062
≥ 30	864 (39.0)	3556 (36.3)	0.056	855 (38.9)	806 (36.7)	0.046
eGFR (mL/min/1.73 m^2^)	40.2 ± 23.4	67.8 ± 25.4	**1.135**	40.2 ± 23.4	66.3 ± 25.1	**1.075**
Any eGFR < 30	2031 (91.8)	0 (0.0)	**4.724**	2016 (91.8)	0 (0.0)	**4.733**
Any eGFR ≥ 30	1734 (78.4)	9296 (95.0)	**0.506**	1720 (78.3)	2118 (96.4)	**0.567**

PSM was performed using clinically relevant baseline covariates, particularly those demonstrating imbalance before matching (absolute SMD > 0.1), including age (continuous), male, Black or African American, hypertensive diseases, disorders of lipoprotein metabolism and other lipidemia, diabetes mellitus, ischemic heart diseases, diseases of arteries, arterioles and capillaries, malignant neoplasm of kidney (except renal pelvis), malignant neoplasm of renal pelvis, malignant neoplasm of ureter, malignant neoplasm of bladder, reduced mobility, overweight and obesity, acquired absence of kidney, antibacterials for systemic use, and proton pump inhibitors. Continuous variables are presented as mean ± SD; categorical variables are presented as n (%). Absolute SMDs > 0.1 are shown in bold. eGFR category rows indicate any recorded value within each range during the 180-day baseline window and are not mutually exclusive. eGFR categories were used for cohort definition but not included as propensity score matching covariates

aCKD, advanced chronic kidney disease; eCKD, early chronic kidney disease; PSM, propensity score matching; BMI, body mass index; eGFR, estimated glomerular filtration rate; SMD, standardized mean difference

PSM was performed using clinically relevant baseline covariates, particularly those demonstrating imbalance before matching (absolute SMD > 0.1), excluding eGFR categories. Following PSM, 2196 patients remained in each cohort. Baseline covariates were well balanced between groups, with all absolute SMDs < 0.1 except for prior antineoplastic radiation therapy, which showed minimal residual imbalance (SMD = 0.104) (Table 1 and Supplementary Table S2).

### Survival analysis of All-cause mortality and irAEs after PSM

**Fig. 2:**
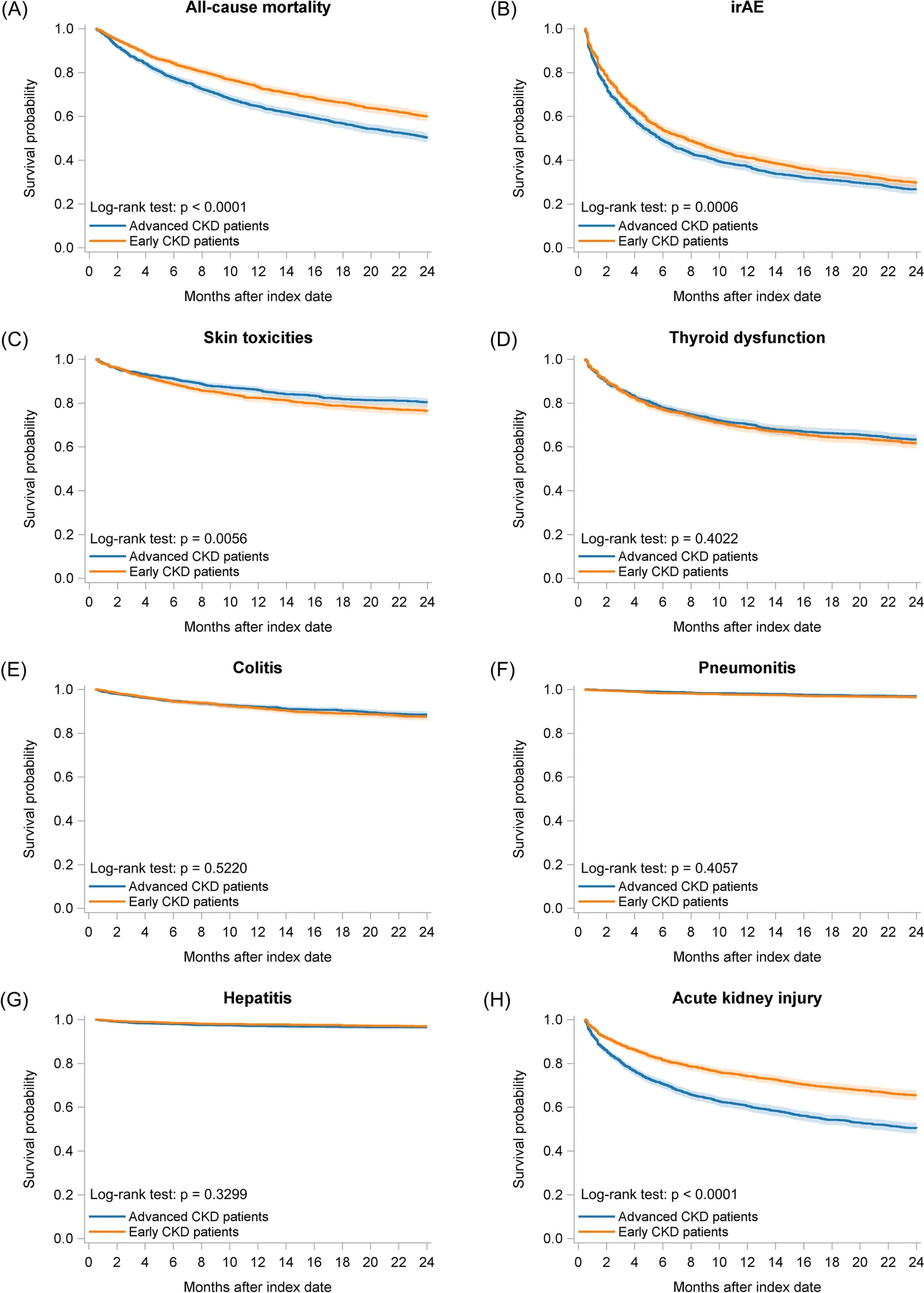
KM curves of all-cause mortality and immune-related adverse events after PSM over two years of follow-up

After PSM, 981 patients (44.7%) in the aCKD group and 779 patients (35.5%) in the eCKD group died during the 2-year follow-up period. Patients with aCKD showed a significantly higher risk of all-cause mortality compared to those with eCKD (HR = 1.372, 95% CI: 1.248–1.507). Regarding irAEs, the aCKD patients demonstrated significantly higher risks of overall irAEs (HR = 1.141, 95% CI: 1.058–1.231) and AKI (HR = 1.662, 95% CI: 1.500–1.840) but lower risk of coded skin toxicities (HR = 0.814, 95% CI: 0.703–0.942) (Table 2). Kaplan–Meier analyses further showed significant differences between groups in all-cause mortality, irAEs, skin toxicities, and AKI throughout the 2-year follow-up period (all log-rank tests: p < 0.05) (Fig. 2). Increased risks of both all-cause mortality and AKI were already evident at 3 months and persisted through 60 months (Supplementary Table S3).

**Table 2:** All-cause mortality and immune-related adverse events after PSM over 2 years of follow-up

	Patients with aCKD (n = 2196)	Patients with eCKD (n = 2196)	HR (95% CI)
All-cause mortality	981 (44.7)	779 (35.5)	**1.372 (1.248–1.507)**
Immune-related adverse events (irAEs)	1367 (62.2)	1320 (60.1)	**1.141 (1.058–1.231)**
Skin toxicities	324 (14.8)	407 (18.5)	**0.814 (0.703–0.942)**
Thyroid dysfunction	640 (29.1)	689 (31.4)	0.955 (0.858–1.064)
Colitis	188 (8.6)	210 (9.6)	0.938 (0.770–1.142)
Pneumonitis	49 (2.2)	60 (2.7)	0.852 (0.584–1.243)
Hepatitis	61 (2.8)	53 (2.4)	1.200 (0.831–1.735)
Acute kidney injury	900 (41.0)	626 (28.5)	**1.662 (1.500–1.840)**

Categorical variables are presented as n (%). Significant results are shown in bold

aCKD, advanced chronic kidney disease; eCKD, early chronic kidney disease; PSM, propensity score matching; HR, hazard ratio; CI, confidence interval

### Age-stratified survival analysis of all-cause mortality and irAEs

Age-stratified subgroup analyses (< 65 vs ≥ 65 years at index) were performed in the pre-matched cohorts using multivariable Cox models adjusted for the same covariates used in PSM. Among patients aged < 65 years, aCKD was associated with higher risks of all-cause mortality (aHR = 1.325) and AKI (aHR = 1.677), but lower risks of skin toxicities (aHR = 0.697) and colitis (aHR = 0.726) during the 2-year follow-up period (all p < 0.05). Among patients aged ≥ 65 years, aCKD was associated with higher risks of all-cause mortality (aHR = 1.378), overall irAEs (aHR = 1.194), and AKI (aHR = 1.627) (all p < 0.001) (Table 3).

**Table 3:** Multivariable Cox proportional hazards analyses in the pre-matched cohorts over 2 years of follow-up, stratified by age at index

aCKD vs eCKD	Overall		Age < 65 years		Age ≥ 65 years	
	aHR (95% CI)	p-Value	aHR (95% CI)	p-Value	aHR (95% CI)	p-Value
All-cause mortality	1.360 (1.265–1.462)	**< 0.001**	1.325 (1.154–1.521)	**< 0.001**	1.378 (1.265–1.501)	**< 0.001**
Immune-related adverse events (irAEs)	1.171 (1.103–1.243)	**< 0.001**	1.096 (0.982–1.224)	0.103	1.194 (1.112–1.282)	**< 0.001**
Skin toxicities	0.835 (0.740–0.942)	**0.003**	0.697 (0.550–0.882)	**0.003**	0.910 (0.785–1.056)	0.216
Thyroid dysfunction	0.964 (0.885–1.051)	0.408	0.960 (0.818–1.127)	0.619	0.987 (0.887–1.097)	0.803
Colitis	0.970 (0.827–1.137)	0.707	0.726 (0.528–0.998)	**0.048**	1.081 (0.892–1.310)	0.425
Pneumonitis	0.920 (0.683–1.241)	0.587	0.942 (0.505–1.757)	0.850	0.942 (0.658–1.346)	0.741
Hepatitis	1.121 (0.843–1.491)	0.431	1.036 (0.595–1.806)	0.900	1.135 (0.812–1.587)	0.458
Acute kidney injury	1.652 (1.529–1.784)	**< 0.001**	1.677 (1.455–1.933)	** < 0.001**	1.627 (1.484–1.785)	**< 0.001**

Multivariable Cox proportional hazards models were performed in the pre-matched cohorts and adjusted for the covariates used for propensity score matching in Table 1 (except the stratified variable). p-Values < 0.05 are shown in bold

aCKD, advanced chronic kidney disease; eCKD, early chronic kidney disease; aHR, adjusted hazard ratio; CI, confidence interval

### Cancer type–specific subgroup analysis (RCC vs UC)

**Fig. 3:**
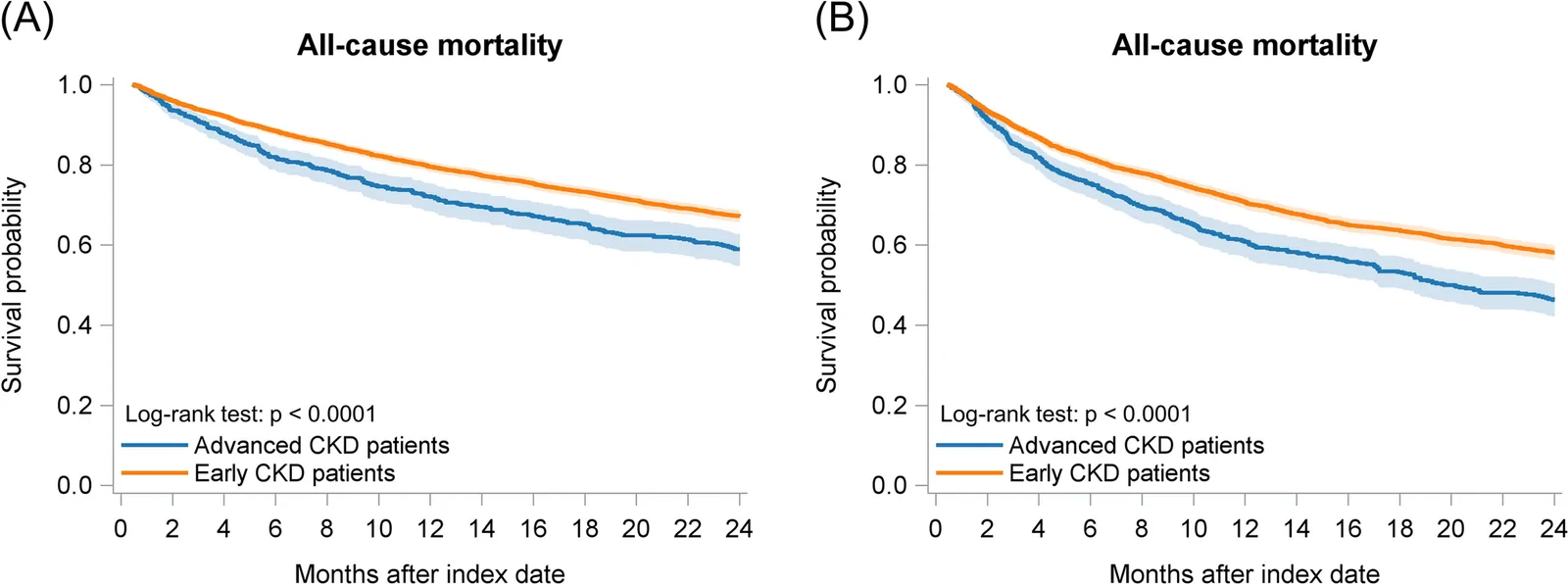
KM curves of all-cause mortality in **A** RCC and **B** UC subgroups

Subgroup analyses were performed to evaluate the association between CKD stage and all-cause mortality according to cancer type. Kaplan–Meier analyses showed significantly higher all-cause mortality among patients with aCKD than those with eCKD in both RCC and UC subgroups (both log-rank tests: p < 0.0001) (Fig. 3). In multivariable Cox models adjusted for the same covariates used in PSM, aCKD remained associated with an increased risk of all-cause mortality in both RCC (aHR = 1.332, 95% CI: 1.183–1.500) and UC (aHR = 1.389, 95% CI: 1.256–1.536) (both p < 0.001).

### Impact of dialysis status on mortality in patients with aCKD

**Fig. 4:**
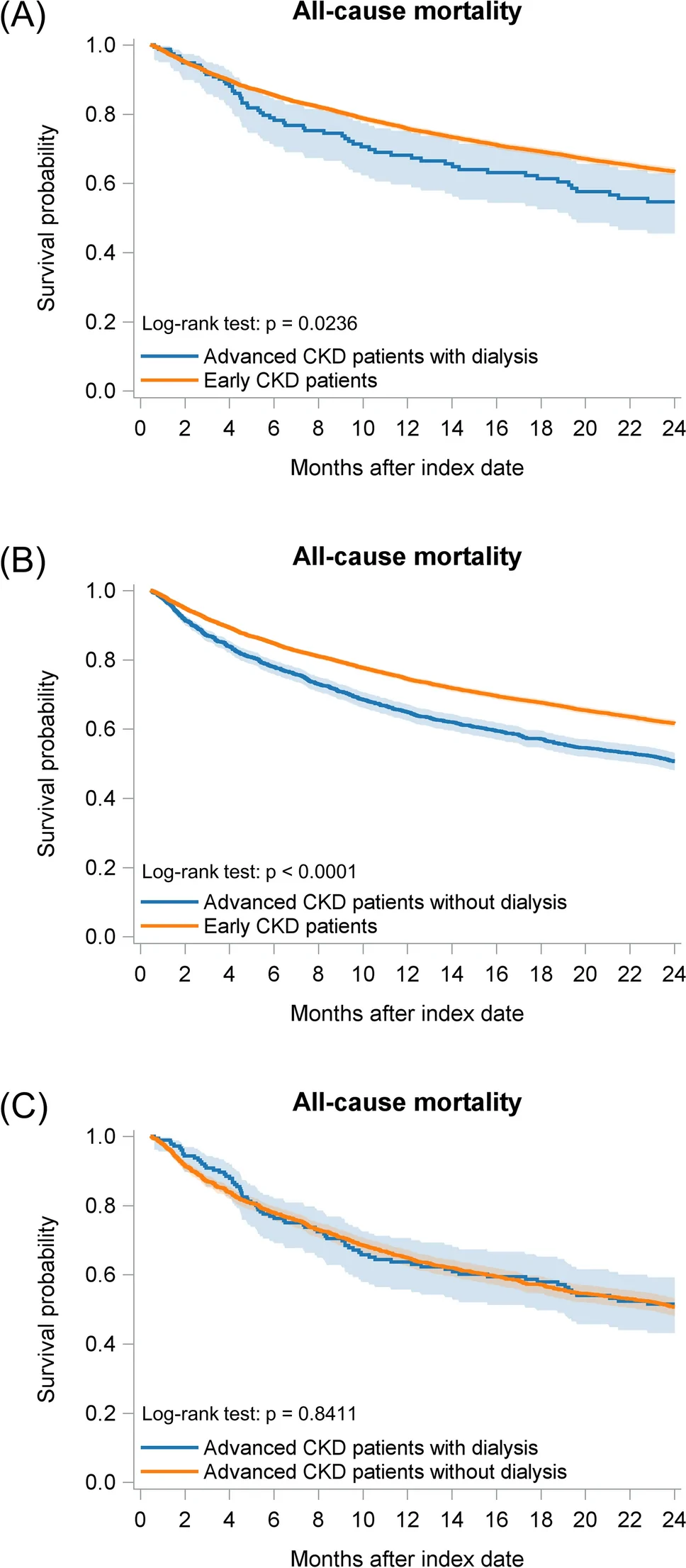
KM curves of all-cause mortality compared between aCKD with dialysis, aCKD without dialysis, and eCKD

Patients with aCKD were stratified according to dialysis status. Kaplan–Meier analyses demonstrated significantly higher all-cause mortality in both aCKD subgroups compared with the eCKD group (log-rank tests: aCKD with dialysis, p = 0.0236; aCKD without dialysis, p < 0.0001), whereas no significant difference was observed between aCKD patients with and without dialysis (Fig. 4). Consistent findings were observed in multivariable Cox models adjusted for the same covariates used in PSM. Compared with the eCKD group, both aCKD patients receiving dialysis (aHR = 1.502, 95% CI: 1.207–1.869, p = 0.0003) and those not receiving dialysis (aHR = 1.345, 95% CI: 1.251–1.446, p < 0.001) had significantly higher risks of all-cause mortality. In contrast, all-cause mortality did not differ significantly between aCKD patients with and without dialysis (aHR = 1.151, 95% CI: 0.914–1.449, p = 0.233).

## Discussion

In this large real-world cohort of patients with RCC or UC and underlying CKD treated with ICIs, we found that aCKD was associated with significantly higher all-cause mortality and a distinct pattern of coded immune-related toxicities. After 1:1 propensity score matching on clinically relevant baseline characteristics, patients with aCKD had a higher 2-year all-cause mortality than those with eCKD, and this increased mortality risk was already evident at 3 months and persisted through 60 months in analyses using different follow-up durations. Patients with aCKD also had a modestly higher risk of overall coded irAEs, mainly driven by a substantially higher risk of AKI. In contrast, thyroid dysfunction, colitis, pneumonitis, and hepatitis did not differ significantly between groups, whereas coded skin toxicities were less frequently observed in the aCKD cohort. This lower rate of coded skin toxicities should be interpreted cautiously because mild cutaneous events may be under-ascertained in ICD-10-CM–based electronic health record data. These findings suggest that aCKD is an important marker of worse prognosis and greater renal vulnerability among ICI-treated patients with genitourinary cancers, while the overall toxicity profile should be interpreted in light of code-based outcome ascertainment and potential residual confounding.

The early and persistent excess mortality observed in the aCKD cohort likely reflects several overlapping mechanisms. Advanced CKD may represent not only impaired renal function but also reduced physiologic reserve, higher cardiovascular and metabolic comorbidity burden, chronic inflammation, malnutrition, and greater susceptibility to treatment-related complications [23]. These factors may increase competing non-cancer mortality and reduce tolerance to systemic cancer therapy [24]. In addition, uremia-related immune dysfunction may impair T-cell activation and proliferation, potentially attenuating the durability of antitumor immune responses to ICIs [25]. Therefore, aCKD should be interpreted as a marker of worse prognosis rather than definitive evidence that CKD severity alone caused the excess mortality.

To date, only three studies, including the present analysis, have evaluated the effectiveness and safety of ICIs in patients with CKD and metastatic genitourinary cancers. Two prior single-center series included 49 patients with CKD (eGFR < 60 mL/min/1.73 m^2^) receiving ICIs for advanced RCC or UC and 41 patients with more advanced CKD (eGFR < 30 mL/min/1.73 m^2^), respectively [10, 11]. Our study, therefore, represents the largest cohort reported so far and extends these observations to a broader, multi-institutional population. Across all three studies, patients with CKD consistently exhibited inferior overall survival compared with those with preserved renal function, underscoring CKD as an important adverse prognostic factor in the ICI era.

### ICI efficacy in older adults with CKD

Older adults, who represent the majority of real-world cancer diagnoses, are consistently underrepresented in clinical trials assessing ICIs; however, age-related immunosenescence might affect ICI efficacy. Although the commonly used age cutoff of 65 years does not fully capture biological aging or frailty, it remains a standard demarcation in several clinical studies. Meta-analyses have indicated that ICI therapies can improve OS and progression-free survival (PFS) in patients below and above 65 years, with no significant disparities [26, 27]. However, ICI effectiveness in older individuals with CKD has not been specifically documented. In the present analysis, aCKD was associated with higher all-cause mortality in both younger and older patients, suggesting that advanced kidney dysfunction may confer adverse prognostic implications across age strata. Nevertheless, older adults with aCKD may remain particularly vulnerable because of frailty, immunosenescence, and competing comorbidity burden, supporting individualized treatment decisions and close multidisciplinary monitoring.

### ICI treatment in patients with aCKD and those undergoing dialysis

Therapeutic management of patients with stage 4–5 CKD, including those undergoing predialysis or dialysis, remains a clinical challenge owing to the absence of established treatment guidelines. Historically, aCKD has been associated with a poor prognosis, primarily owing to decreased physical performance and an elevated cancer progression risk. Managing adverse events from ICIs introduces further complexity, particularly in non-dialyzed patients with unstable renal function, compared with those receiving hemodialysis.

ICIs are predominantly excreted through non-renal pathways as opposed to hemodialysis because of their high molecular weights. This pharmacokinetic characteristic negates the need for dosage adjustments in patients undergoing dialysis. A 2021 literature review [12] reported that among 80 patients with ESKD treated with ICIs, none required dose modifications, and the incidence of irAEs paralleled that reported in the general ICI-treated population. Nonetheless, the overall effectiveness of ICIs may be compromised in patients with ESRD owing to diminished immune cell functionality.

Although we noted increased all-cause mortality rates in both patients with aCKD undergoing dialysis and those in the pre-dialysis phase compared with the eCKD cohort, no statistically significant difference was observed in the mortality rates between the subgroups of aCKD. Moreover, only 135 patients on dialysis (approximately 7.5% of the total CKD cohort) received ICIs. This small proportion of patients highlights the need for further research to better understand the implications of ICI therapy in this subgroup.

### irAEs in CKD

Our analysis highlights both the usefulness and limitations of code-based irAE ascertainment in large EHR databases. Skin toxicities are often reported as among the most frequent irAEs during clinical trials, occurring in over one-third of patients [28]. However, coded skin toxicities were less frequently observed in patients with aCKD than in those with eCKD in our study. This finding should not be interpreted as a protective effect of aCKD. Mild cutaneous events, such as rash, pruritus, or dermatitis, may be documented in clinical notes without being assigned specific ICD-10-CM diagnosis codes. Therefore, the lower observed rate of coded skin toxicities in the aCKD cohort may reflect under-ascertainment or differences in documentation and coding practices rather than a truly lower clinical incidence.

AKI was the most prominent safety signal in the aCKD cohort. Previous reviews have shown that AKI is a recognized complication among ICI-treated patients, although biopsy-confirmed ICI-induced AKI represents only a subset of all AKI events [18, 29]. In the present study, patients with aCKD had a substantially higher risk of coded AKI than those with eCKD; however, AKI codes should be interpreted broadly and do not necessarily represent biopsy-confirmed ICI-induced nephritis. In patients with aCKD, AKI may also reflect hemodynamic instability, infection, nephrotoxic medication exposure, contrast exposure, obstructive uropathy, cancer progression, or hospitalization-related events [30]. Therefore, the higher AKI risk observed in aCKD patients should be viewed as a signal of renal vulnerability requiring close monitoring, rather than definitive evidence of excess immune-mediated nephrotoxicity.

Previous studies focusing on metastatic genitourinary cancers have suggested that CKD does not inherently increase the irAE risk [10, 11], highlighting the necessity for cautious interpretation and further research to explore the interactions between CKD and irAEs in patients undergoing ICI therapy.

### Limitations

Our study has several limitations. First, despite matching on multiple baseline characteristics, including demographics, comorbidities, medication use, cancer-related treatment history, and health care utilization, the retrospective design remains subject to selection and information biases. Detailed tumor stage, metastatic burden, formal ICI line of therapy, treatment intensity, and corticosteroid indication, dose, duration, and cumulative exposure were unavailable; therefore, residual confounding by these unmeasured factors cannot be excluded. Second, due to the lack of specific ICD-10-CM codes, we relied on previously published code algorithms to identify AKI and other irAEs. While our estimated irAE incidences were broadly comparable to those reported in prior studies, code-based definitions are necessarily broad and may misclassify non–ICI-related events as irAEs or fail to capture milder events that are not coded, particularly mild cutaneous toxicities such as rash, pruritus, or dermatitis, leading to both false positives and false negatives. Third, because we excluded patients who died or experienced an irAE within 14 days of ICI initiation, our analyses estimate outcomes only among patients who survived this initial period. This design may lead to underestimation of very early mortality and toxicities, especially in patients with aCKD. In addition, because TriNetX categorical laboratory indicators are not mutually exclusive when multiple laboratory values occur within the baseline window, eGFR category rows should be interpreted as indicators of any recorded value within each range rather than mutually exclusive renal function strata. Finally, limited information on irAE grading, management, and downstream clinical outcomes hindered a more detailed evaluation of irAE severity and its implications for treatment decision-making and prognosis.

## Conclusions

Our study offers important real-world insights into ICI use for advanced genitourinary cancers in patients with CKD, particularly those with an eGFR < 30 mL/min/1.73 m^2^. Although aCKD was associated with worse overall survival and a modestly higher risk of overall coded irAEs, this excess toxicity signal was mainly driven by AKI rather than consistent increases across all organ-specific irAEs. These findings suggest that CKD alone should not automatically preclude ICI use in carefully selected patients, but patients with aCKD require close renal monitoring and multidisciplinary management throughout treatment. Prospective studies with detailed oncologic and toxicity assessments are needed to confirm these observations and to better inform future treatment algorithms for this high-risk population.

## Supplementary Information

Below is the link to the electronic supplementary material.

Supplementary file1 (DOCX 27 KB)

## Data Availability

This study was based on data from the TriNetX database, which includes de-identified patient information. Access to these data is governed by the TriNetX platform and is available only under specific data use agreements. Thus, the authors are unable to directly share the raw data used for this analysis. However, researchers interested in accessing the data may submit a request directly through the TriNetX network, following TriNetX's data access policies and requirements for protocol submission. Any inquiries or protocols for re-analysis should be directed to TriNetX for approval. The study protocol and statistical analysis plan can be shared upon reasonable request to the corresponding author.
