# Supplementary material for: Efficacy of checkpoint inhibitors in genitourinary cancers with advanced chronic kidney disease
Source: Cancer Immunol Immunother. 2026 Jul 10;75(10):245. doi: 10.1007/s00262-026-04482-0 (PMC13638894; doi:10.1007/s00262-026-04482-0)
Supplement: Supplementary file 1 [file 262_2026_4482_MOESM1_ESM.docx]

**Supplementary Tables**

**Supplementary Table S1**. Immunological checkpoint inhibitor (ICI) codes and immune-related adverse event codes

 Prescribed ICI at least once after 1^st^ RCC/UC diagnosis:

Pembrolizumab: 【RxNorm=1547545, HCPCS= J9271】

Nivolumab: 【RxNorm=1597876, HCPCS = J9299, J9298】

Durvalumab: 【RxNorm=1919503, HCPCS = J9173】

Atezolizumab: 【RxNorm=1792776, HCPCS = J9022】

Cemiplimab: 【RxNorm=2058826, HCPCS = J9119】

Avelumab: 【RxNorm=1875534, HCPCS = J9023】

 Immune-related adverse events (irAEs)
• skin toxicities: R21 or L80
• thyroid dysfunction: E03.2, E03.8, E03.9, or E05
• colitis: K52.3, K52.8, or K52.9
• pneumonitis: J70.2, J70.3, J70.4, J70.8, J84.11, or J84.9
• hepatitis: K71, K75.4, or K75.9
• acute kidney injury: N17.8, N17.9, N19, N99.0

**Supplementary Table S2**. Additional baseline characteristics of the study population before and after PSM

|  | **Before PSM** | | | **After PSM** | | |
| --- | --- | --- | --- | --- | --- | --- |
|  | **Patients with aCKD (n = 2,213)** | **Patients with eCKD (n = 9,784)** | **SMD** | **Patients with aCKD (n = 2,196)** | **Patients with eCKD (n = 2,196)** | **SMD** |
| **Cancer-related factors before ICI initiation, n (%)** |  |  |  |  |  |  |
| Prior chemotherapy-related encounter | 461 (20.8) | 2044 (20.9) | 0.001 | 458 (20.9) | 520 (23.7) | 0.068 |
| Prior radiation oncology treatment | 265 (12.0) | 1341 (13.7) | 0.052 | 263 (12.0) | 315 (14.3) | 0.070 |
| Prior immunotherapy-related encounter | 226 (10.2) | 1023 (10.5) | 0.008 | 224 (10.2) | 240 (10.9) | 0.024 |
| Palliative care encounter | 161 (7.3) | 562 (5.7) | 0.062 | 158 (7.2) | 144 (6.6) | 0.025 |
| Prior antineoplastic radiation therapy | 60 (2.7) | 419 (4.3) | 0.086 | 60 (2.7) | 103 (4.7) | **0.104** |
| **Performance status, n (%)** | (0.0) |  |  |  |  |  |
| Reduced mobility | 83 (3.8) | 179 (1.8) | **0.117** | 74 (3.4) | 87 (4.0) | 0.032 |
| Dependence on supplemental oxygen | 44 (2.0) | 144 (1.5) | 0.040 | 44 (2.0) | 44 (2.0) | 0 |
| Difficulty in walking, not elsewhere classified | 29 (1.3) | 95 (1.0) | 0.032 | 29 (1.3) | 31 (1.4) | 0.008 |
| Dependence on wheelchair | 10 (0.5) | 28 (0.3) | 0.027 | 10 (0.5) | 10 (0.5) | 0 |
| Unspecified symptoms and signs involving the nervous and musculoskeletal systems | 10 (0.5) | 17 (0.2) | 0.050 | 10 (0.5) | 10 (0.5) | 0 |
| Dependence on respirator | 10 (0.5) | 22 (0.2) | 0.039 | 10 (0.5) | 10 (0.5) | 0 |
| Limitation of activities due to disability | 10 (0.5) | 10 (0.1) | 0.067 | 10 (0.5) | 0 (0.0) | 0.096 |
| **Lifestyles, n (%)** |  |  |  |  |  |  |
| Nicotine dependence | 301 (13.6) | 1400 (14.3) | 0.020 | 297 (13.5) | 343 (15.6) | 0.059 |
| Tobacco use | 101 (4.6) | 392 (4.0) | 0.028 | 98 (4.5) | 104 (4.7) | 0.013 |
| Alcohol related disorders | 43 (1.9) | 221 (2.3) | 0.022 | 42 (1.9) | 45 (2.0) | 0.010 |

aCKD, advanced chronic kidney disease; eCKD, early chronic kidney disease; PSM, propensity score matching; SMD, standardized mean difference

Continuous variables are presented as mean ± SD; categorical variables are presented as n (%).

Absolute SMDs > 0.1 are shown in bold.

**Supplementary Table S3**. All-cause mortality and immune-related adverse events after PSM at different follow-up durations

|  | **HR (95% CI)** | | | | |
| --- | --- | --- | --- | --- | --- |
|  | **3 months** | **6 months** | **12 months** | **36 months** | **60 months** |
| **aCKD vs eCKD** | **n = 2,244 pairs** | **n = 2,221 pairs** | **n = 2,244 pairs** | **n = 2,244 pairs** | **n = 2,244 pairs** |
| **All-cause mortality** | **1.294 (1.081 – 1.548)** | **1.401 (1.220 – 1.608)** | **1.315 (1.180 – 1.465)** | **1.283 (1.176 – 1.401)** | **1.268 (1.166 – 1.379)** |
| **Immune-related adverse events (irAEs)** | **1.266 (1.139 – 1.408)** | **1.210 (1.108 – 1.322)** | **1.211 (1.119 – 1.312)** | **1.203 (1.117 – 1.295)** | **1.199 (1.114 – 1.291)** |
| Skin toxicities | 0.910 (0.712 – 1.164) | **0.755 (0.621 – 0.919)** | **0.822 (0.699 – 0.968)** | **0.834 (0.724 – 0.961)** | **0.849 (0.739 – 0.975)** |
| Thyroid dysfunction | 1.070 (0.910 – 1.259) | 1.070 (0.937 – 1.221) | 1.060 (0.944 – 1.190) | 1.045 (0.940 – 1.162) | 1.046 (0.942 – 1.161) |
| Colitis | 0.996 (0.698 – 1.423) | 1.011 (0.774 – 1.321) | 1.005 (0.802 – 1.258) | 1.022 (0.846 – 1.235) | 1.032 (0.861 – 1.238) |
| Pneumonitis | 1.263 (0.591 – 2.699) | 0.860 (0.515 – 1.436) | 0.843 (0.555 – 1.280) | 0.876 (0.620 – 1.239) | 0.895 (0.646 – 1.239) |
| Hepatitis | 1.654 (0.934 – 2.927) | 1.042 (0.677 – 1.602) | 1.236 (0.835 – 1.829) | 1.149 (0.803 – 1.644) | 1.142 (0.802 – 1.624) |
| Acute kidney injury | **1.826 (1.555 – 2.143)** | **1.741 (1.530 – 1.980)** | **1.742 (1.559 – 1.947)** | **1.637 (1.485 – 1.804)** | **1.602 (1.457 – 1.761)** |

aCKD, advanced chronic kidney disease; eCKD, early chronic kidney disease; HR, hazard ratio; CI, confidence interval

Due to TriNetX built-in functionality, analyses at different follow-up periods were conducted separately, with propensity score matching performed for each follow-up window.

Significant results are shown in bold.
